# Comprehensive biological evaluation of infuzide as a potent antimicrobial, alone and in combination with gentamicin, linezolid, and minocycline targeting MDR *Staphylococcus aureus* and *Enterococcus* sp.

**DOI:** 10.1128/spectrum.00279-25

**Published:** 2025-06-02

**Authors:** Rahul Maitra, Deepanshi Saxena, Abdul Akhir, Anna Kapusterynska, Michel Baltas, Sidharth Chopra

**Affiliations:** 1 Division of Molecular Microbiology and Immunology, CSIR-Central Drug Research Institute 682841 , Lucknow, Uttar Pradesh, India; 2 CNRS, Laboratoire de Chimie de Coordination (LCC) Inserm ERL 1289, Université de Toulouse, UPS, INPT 27091, Toulouse, Occitanie, France; 3 Academy of Scientific and Innovative Research (AcSIR) 550336, Ghaziabad, Uttar Pradesh, India; Houston Methodist, Houston, Texas, USA

**Keywords:** antimicrobial agents, *Staphylococcus aureus*, mechanochemical

## Abstract

Antimicrobial resistance is the greatest threat to modern healthcare systems. The unrelentingly increasing drug resistance manifests in ESKAPE pathogens, thus necessitating urgent novel drug discovery. Whole cell-screening assays were used to identify Infuzide, expressing potent antimicrobial activity against gram-positive pathogens *Staphylococcus aureus* and *Enterococcus*. To probe if Infuzide’s inactivity against gram-negative pathogens was due to entry issues, its activity was determined in the presence of Polymyxin B nonapeptide, a known membrane permeabilizer. Furthermore, its activity against clinical drug-resistant strains was evaluated. As the next step, the compound’s time-kill kinetics against *S. aureus* was determined, along with its ability to synergize with approved drugs via checkerboard assays; this was further validated via combination time-kill kinetics assays. Infuzide’s *in vitro* post-antibiotic effect, ability to eradicate *S. aureus* biofilms, and ability to isolate resistant mutants were also ascertained. Eventually, Infuzide’s *in vivo* efficacy alone and in combination was assessed in murine neutropenic thigh and skin infection model against drug-susceptible and drug-resistant *S. aureus*. Infuzide exhibits highly potent activity against *S. aureus* and *Enterococcus*, which is comparable with standard of care. In addition, infuzide expresses concentration-dependent bactericidal activity with ~5.9 log_10_cfu/mL reduction within 6 h of treatment and synergizes with gentamicin, linezolid, and minocycline, even against MDR strains. Infuzide also inhibits bacterial growth in both biofilm and intracellular phases and exhibits robust *in vivo* efficacy in both neutropenic thigh and murine skin infection models without inducing resistance. Taken together, Infuzide exhibits all characteristics required for pre-clinical development as an anti-staphylococcal compound.

**IMPORTANCE:**

This study identifies Infuzide as a novel antimicrobial against *S. aureus.*

## INTRODUCTION

Antimicrobial resistance (AMR) is the leading cause of mortality worldwide and is responsible for almost ~10% of global deaths (1). Most of these infections and consequently deaths are attributable to the so-called ESKAPE pathogens consisting of *Enterococcus* sp, *Staphylococcus aureus*, *Klebsiella pneumoniae*, *Acinetobacter baumannii*, *Pseudomonas aeruginosa,* and *Enterobacter* sp (2). A striking feature of the ESAKPE pathogens is the presence of two World Health Organization (WHO) high-priority pathogens, that is, *S. aureus* and *Enterococcus* sp. Both of these pathogens are the leading causes of hospital-acquired infections (HAI) as well as community-acquired infections (CAI), leading to significant morbidity and mortality (3). *S. aureus* causes skin and soft tissue infections, osteoarticular infection, bloodstream infections, pneumonia, infective endocarditis, and device-related infections (4). Methicillin-susceptible *S. aureus* can be successfully treated with β-lactams, but methicillin-resistant *S. aureus* (MRSA) is resistant to β-lactams and consequently much harder to treat (5). As a result of the unhindered expansion of various MRSA clones worldwide, there is much more reliance on vancomycin, which has become the standard of care (SoC), leading to the generation of vancomycin-intermediate resistant *S. aureus* (VISA) and vancomycin-resistant *S. aureus* (VRSA) (6). Another WHO high-priority pathogen is *Enterococcus* that frequently inhabits human gastrointestinal, vaginal tracts, as well as oral cavity, with the most common species being *Enterococcus faecalis* and *E. faecium* and is responsible for causing wound and soft-tissue infections, urinary tract infections, meningitis, bacteremia, sepsis, biofilm-associated infections of medical devices, and endocarditis with an estimated ~250,000 deaths in 2019 (1). Like *S. aureus*, *Enterococcus* also displays a high propensity to develop resistance to clinically utilized antibiotics, especially vancomycin, thus posing a severe challenge to infection control. Vancomycin-resistant *Enterococcus* (VRE) is often associated with HAI, and the most recent estimate is ~30% in 2019 (7). Prevalence of MRSA has increased by ~50%–60%, whereas VRSA increased by 3.5-fold between 2006 and 2020 (8). In view of this increased number of multi-drug resistance (MDR) exhibited by both *S. aureus* as well as *Enterococcus* sp., it necessitates novel drug discovery acting via previously untargeted pathways to lessen the inductions and development of AMR.

In this context, several scaffolds and their derivatives have been already reported against drug-resistant *S. aureus* (9–12). Hydrazone compounds represent an important class of compounds, potentially exhibiting biological properties, that is, antimicrobial (13), anti-inflammatory (14), anti-infective (15), and antileishmanial (16). On the other hand, for two decades, new approaches in the area of organic synthesis oriented toward biologically active compounds integrate the need for efficient and environmentally safe methods. In that respect, mechanochemistry has rapidly emerged as a powerful tool enabling environmentally benign and sustainable synthesis (17). Several years ago, we launched a research program focused on the mechanochemical synthesis of hydrazones and derivatives within potential biological activities (18). Very recently, we reported the construction by mechanochemical means and the biological activities of a series of hydrazones obtained by coupling hydrazines with vanillin and furanyl aldehydes (12). Particularly, the furanyl fragment is present in various active pharmaceutical ingredients (API) hydrazones, like the anti-infective nifroxazide and the antibacterial and antiprotozoal furazolidone (19).

In our recent paper on hydrazones, we have reported the synthesis of a series of 17 hydrazones obtained in quantitative yields under solvent-free mechanochemical conditions and their corresponding antibacterial activities. The coupling proceeded in a planetary ball mill Pulverisette 7 (P7) (Fritsch) between the corresponding aldehydes and heterocyclic hydrazines or hydrazinamides in short reaction times. Among the 17 compounds studied (synthesis and biological activities), one compound, never obtained before, namely infuzide (*3-(1H-indol-2-yl)-N'-[(1E,2E)−3-(5-nitrofuran-2-yl)prop-2-en-1-ylidene]acetohydrazide*) was identified as a highly potent hit molecule selectively targeting gram-positive human pathogens *S. aureus* and *Enterococcus* sp (12). In the present work, we present the detailed biological evaluation of its antimicrobial potential, including in various *in vivo* murine models, further highlighting its capacity to be considered as a lead molecule for the treatment of serious infections caused by WHO high-priority pathogens.

## RESULTS AND DISCUSSION

### Laboratory scale synthesis and chemical verification of Infuzide

**Fig 1:**
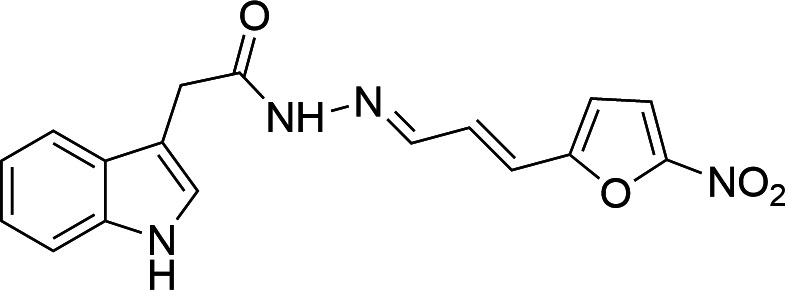
Structure of Infuzide (C_17_H_14_N_4_O_4_).

The laboratory-scale synthesis and chemical verification of Infuzide (Fig. 1) was carried out as described earlier (12). The stability of the compound was checked by analytical reverse-phase chromatography. A solution of Infuzide (3 mg) in MeOH + 0.1% TFA (0.5 mL) was injected at T = 0 min, T = 36 h, and T = 72 h in an XBridge C18 5 µm column. Infuzide remained unchanged, thus showing excellent stability under these conditions (Fig. S1).

### Infuzide is potently active against *S. aureus* and *Enterococcus* sp. and exhibits minimal toxicity against Vero cells and no hemolytic potential

Infuzide exhibits selective activity against gram-positive bacteria (GPB) *S. aureus* ATCC 29213 (MIC 1 µg/mL) and *Enterococcus faecium* NR 31912 (2 μg/mL) (Table 1). Infuzide was inactive against gram-negative bacterial (GNB) pathogens tested except against *E. coli* ATCC 25922, where it exhibited moderate activity (MIC 16 µg/mL). This activity against GPB is quite comparable with that of vancomycin, which is the SoC for infections caused due to these pathogens (Table 1). Thus, Infuzide is selectively active against GPB human pathogens and lacks activity against GNB pathogens with the exception of moderate antimicrobial activity against *E. coli*. In the next step, the minimum bactericidal concentration (MBC) was determined to be 1 µg/mL along with levofloxacin and linezolid as comparator, and data are shown in Table 1. As can be seen, the MIC/MBC ratio for Infuzide is 1; thus, it acts in a cidal manner, whereas for linezolid, the ratio is >8, thus acting as a static drug.

**TABLE 1:** MIC (µg/mL), minimum bactericidal concentration (MBC) (µg/mL), and selectivity index (SI) of selected hydrazones against the human pathogen panel^*a*^

Compound	MIC (µg/mL)						CC_50_ (µg/mL) against Vero cells	SI for *S. aureus* (CC_50_/MIC)	HC_50_ (µg/mL) against human RBC	MBC (µg/mL)	MIC/MBC
	*S. aureus* ATCC 29213	*E. faecium* NR 31912	*E. coli* ATCC 25922	*K. pneumoniae* BAA 1705	*A. baumannii* BAA 1605	*P. aeruginosa* ATCC 27853					
Infuzide	1	2	16	>64	>64	>64	10	10	>100	1	Cidal
Levofloxacin	0.062	>64	0.003	64	4	0.5	NT	NT	NT	1	Cidal
Vancomycin	1	>64	NT	NT	NT	NT	NT	NT	NT	NT	NT
Linezolid	2	NT	NT	NT	NT	NT	NT	NT	NT	>16	Static

^*a*^ CC50, concentration to kill 50% of the Vero cells; NT, not tested.

Hypothetically, the absence of activity against GNB could potentially be due to the presence of an intact outer membrane (OM), a well-recognized permeability barrier (20). To test this hypothesis, we determined the MIC of Infuzide in the presence of polymyxin B nonapeptide (PMBN), a delipidated version of polymyxin B, which is designed to increase the permeability, without expressing any antimicrobial activity, and the data are shown in Table 2 (21). In the presence of PMBN, Infuzide was 8-fold more potent against *E. coli* ATCC 25922 (MIC dropped from 16 μg/mL to 2 μg/mL). A similar pattern was observed against *A. baumannii* BAA-1605 (MIC dropped from being inactive to 32 µg/mL), thus confirming our hypothesis. Both the positive controls, vancomycin and rifampicin, exhibited a significant drop in MIC due to a permeabilized OM, thus validating the assay, whereas levofloxacin, as the negative control, performed as expected, with no change in MIC.

**TABLE 2:** MIC (µg/mL) of infuzide against select gram-negative pathogens in the presence of PMBN

Bacterial strain	MIC (μg/mL)											
	PMBN^*a*^	Infuzide			Rifampicin			Vancomycin			Levofloxacin	
		PMBN		Fold change in MIC	PMBN		Fold change in MIC	PMBN		Fold change in MIC	PMBN	
		(−)	(+)		(−)	(+)		(−)	(+)		(−)	(+)
*E. coli* ATCC 25922	>64	16	2	8	4	0.0625	64	512	64	8	0.0312	0.015
*A. baumannii* BAA-1605	>64	>64	32	~4	4	0.0625	64	128	32	4	8	8

^*a*^ At 10 µg/mL.

The selectivity index of Infuzide was determined against Vero cells to be 10 (Table 1), which is suitable to be progressed into pre-clinical testing. Additionally, the hemolytic potential of Infuzide was determined against human RBCs, and the HC_50_ was determined to be >100 µg/mL with <2% hemolysis at 100 µg/mL, which is 100× MIC (Table 1 and Fig S2). Taken together, Infuzide is non-toxic and non-hemolytic to eukaryotic cells with exquisite specificity for bacterial cells.

### Infuzide is highly efficacious against clinical, multi-drug resistant (MDR) *S. aureus* and *Enterococcus,* including VRSA and VRE

One of the most critical tests of any new molecule under pre-clinical development as an antibacterial is to exhibit as little variation in MIC as possible when tested against clinical, pathogenic MDR isolates. In order to determine the potential activity against MDR clinical pathogens, Infuzide was tested against a multiple-strain panel consisting of 10 MRSA, 3 VRSA, and 8 *Enterococcus,* including 3 VRE, and the data are shown in Table 3. Infuzide impressively exhibited equi-potent activity against MRSA, VRSA, and *Enterococcus* sp., including VRE, even in the presence of a wide variety of antimicrobial resistance mechanisms, differing sites and year of isolation, and presence of several different virulence factors. This ability of Infuzide to exhibit equi-potent activity against a clinical MDR panel indicates that it targets a potentially novel target, not currently targeted by any FDA-approved drug, and additionally, it is not affected by the presence of varied antimicrobial resistance mechanisms expressed by these strains.

**TABLE 3:** MIC (µg/mL) of Infuzide against clinical, drug-resistant *S. aureus* and *Enterococcus* sp

Strain	Resistant to antibiotics:	Molecular details	MIC (µg/mL) of Infuzide
MSSA			
ATCC 29213	None	Type strain	1
MRSA			
NRS100	Methicillin, tetracycline	Contains subtype I *mec* cassette and large variety of virulence factors, NorA efflux and EmrB/QacA positive strain	0.5
NRS119	Methicillin, gentamicin, linezolid, trimethoprim/sulfamethoxazole	Contains subtype IV *mec* cassette and G2576T mutation in domain V in one or more 23S rRNA genes	1
NRS129	Chloramphenicol	*mecA* negative	0.5
NRS186	Methicillin, levofloxacin, meropenem	USA 300 type CA-MRSA, PVL virulence factor positive and contains *mec* type IV cassette	1
NRS191	Methicillin, levofloxacin, meropenem	USA 600 type CA-MRSA, PVL virulence factor negative and contains *mec* type II cassette	1
NRS192	Methicillin, levofloxacin, meropenem, erythromcyin	CA-MRSA, PVL virulence factor negative and contains *mec* type II cassette	1
NRS193	Methicillin, levofloxacin, meropenem	CA-MRSA, PVL factor negative and contains *mec* type II cassette	1
NRS194	Methicillin, meropenem	CA-MRSA, PVL virulence factor positive and contains *mec* type V cassette	1
NRS198	Methicillin, levofloxacin, meropenem	USA 100 type CA-MRSA, PVL virulence factor negative and contains *mec* type II cassette	1
VRSA			
VRS 1	Methicillin, levofloxacin, meropenem, vancomycin, gentamicin, teicoplanin, spectinomycin	USA 100, contains *mec* subtype II cassette and *vanA,* negative for *vanB*, *vanC1*, *vanC2*, *vanD*, *vanE*, PVL and ACME, NorA and norB efflux positive strain	1
VRS 4	Methicillin, levofloxacin, meropenem, vancomycin, gentamicin, teicoplanin, spectinomycin	USA 100, contains mec subtype II cassette and *vanA,* negative for *vanB*, *vanC1*, *vanC2*, *vanD*, *vanE*, PVL and ACME, NorA and NorB efflux positive strain	1
VRS 12	Methicillin, levofloxacin, meropenem, vancomycin, gentamicin, teicoplanin, spectinomycin	Data not available	1
*Enterococcus*			
*E. faecalis* NR 31884	Gentamicin	A hemolytic, cytolytic isolate strain B3119 was isolated in the USA from human blood in 1987	0.5
*E. faecalis* NR 31885	High level resistance to gentamicin	Cytolytic isolate, strain B3196 was isolated in the USA from human blood in 1987	0.5
*E. faecalis* NR 31886	High level resistance to gentamicin	A hemolytic isolate, B3286 was isolated from human blood in 1987 in the USA	0.5
*E. faecalis* NR 31887	High level resistance to gentamicin	B3336 is an infectious clinical isolate collected from human blood in 1987 in the USA	0.5
*E. faecalis* NR 31888	High level resistance to gentamicin	Cytolytic isolate B4008 was isolated from human blood in 1987 in the USA	0.5
Vancomycin-resistant enterococci (VRE)			
*E. faecium* NR 31903	Vancomycin, minocycline, levofloxacin	EnGen0314 was isolated from the stool of a human patient prior to bacteremia	2
*E. faecium* NR 31909	Vancomycin, minocycline, levofloxacin	EnGen0316 was isolated from the stool of a human patient prior to bacteremia	4
*E. faecium* NR 31912	Vancomycin, minocycline, levofloxacin	EnGen0312 was isolated from the stool of a human patient having dominance of vancomycin-resistant *Enterococcus* in stool but no bacteremia	2

### Infuzide expresses concentration-dependent time-kill kinetics against *S. aureus*

**Fig 2:**
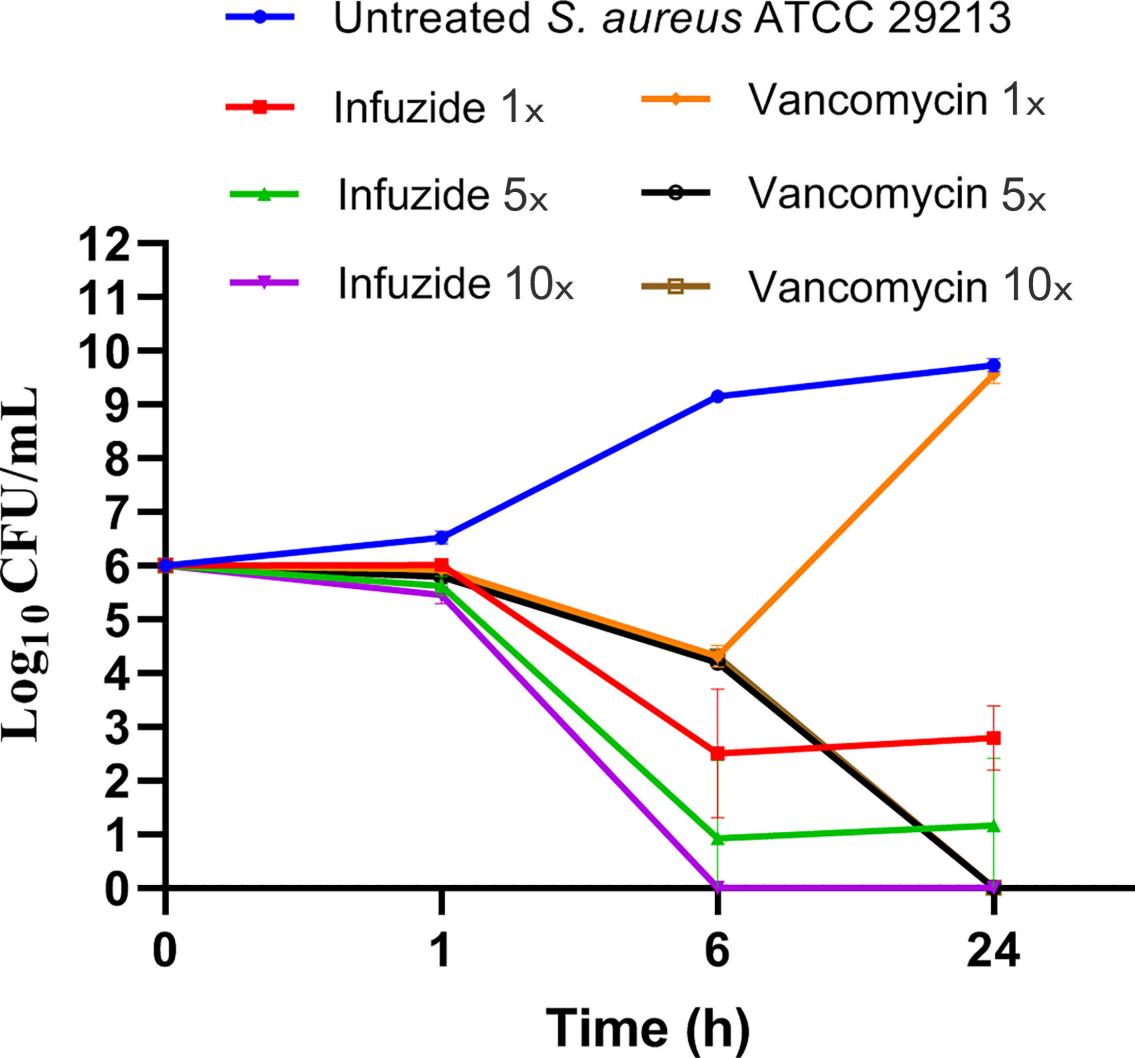
Time-kill kinetics of Infuzide and vancomycin against *S. aureus* ATCC 29213 at various time points and multiple concentrations. Each experiment was performed in triplicate, and the entire experiment was repeated twice. The average values are provided with standard deviations wherever applicable. * Indicates *P* ≥ 0.5, ** indicates *P* < 0.05, and *** indicates *P* < 0.005.

In the next step, the time-kill kinetics of Infuzide was determined along with vancomycin as control against *S. aureus* ATCC 29213 at various time points and multiple concentrations. This important pre-clinical parameter is designed to distinguish between bacteriostatic and bactericidal action and in turn influences the dosing regimen. The data are plotted in Fig. 2 and clearly demonstrate that infuzide exhibits classical concentration-dependent time-kill kinetics with ~3.2 log_10_ cfu/mL reduction at 1× MIC at 24 h and ~5.9 log_10_ cfu/mL reduction at 10× MIC at 6 h with no regrowth till 24 h. In comparison, vancomycin was barely effective at 1× MIC and reduced ~6.0 log_10_ cfu/mL at 10× MIC in 24 h. The kill kinetics exhibited by Infuzide are comparatively much faster with greater reduction in cfu/mL than those exhibited by vancomycin and demonstrate a concentration-dependent decrease, a feature not exhibited by vancomycin. This also matches with the MBC exhibited by Infuzide (Table 1). Taken together, Infuzide exhibits rapid, concentration-dependent bactericidal activity, which is better than vancomycin against *S. aureus* ATCC 29213.

### Infuzide displayed a superior post-antibiotic effect (PAE) against *S. aureus*

The post-antibiotic effect or PAE is an important pharmacokinetic parameter influencing dosing as well as directly impacting the generation of resistance. To assess the PAE of infuzide, the time for increase in 1 log_10_ CFU/mL of bacterial culture was analyzed and is displayed in Table 4. Infuzide displayed a significant inhibition of bacterial growth after compound removal, with time taken for 1 log_10_ CFU/mL increase in the bacterial growth of 7 h at 5× and 10× MIC, which is superior to that exhibited by vancomycin (2.5 h) and levofloxacin (2.5 h) at 5× and 10× MIC, respectively. Thus, Infuzide expresses a superior PAE compared with SoC vancomycin and levofloxacin.

**TABLE 4:** Post-antibiotic effect of infuzide compared with vancomycin and levofloxacin at different MIC

Condition	Time for 1 log_10_ increase in bacterial CFU count (h)	PAE (h)
Untreated	~2.5	0
Infuzide 1× MIC	~5	~2.5
Infuzide 5× MIC	~9.5	~7
Infuzide 10× MIC	~9.5	~7
Vancomycin 1× MIC	~5	~2.5
Vancomycin 5× MIC	~5	~2.5
Vancomycin 10× MIC	~5	~2.5
Levofloxacin 1× MIC	~3	~0.5
Levofloxacin 5× MIC	~4	~1.5
Levofloxacin 10× MIC	~4	~1.5

### Infuzide synergizes with protein synthesis inhibitors against *S. aureus*

Multi-drug regimen is the preferred choice for the treatment of bacterial infections as it has been shown to retard the development of drug resistance as well as lead to a faster clearance of infection. In this context, the ability of infuzide to interact with various drugs utilized for the treatment of staphylococcal infections was checked via the checkerboard method, and the results are tabulated in Table 5. Infuzide clearly synergizes with linezolid and shows partial synergy with gentamicin and minocycline, while with all other antimicrobials tested, it does not interact at all. Intriguingly, all synergizing antibiotics are protein synthesis inhibitors, with linezolid inhibiting the 50S subunit of the ribosome and gentamicin and minocycline inhibiting the 30S subunit.

**TABLE 5:** Drug interaction of Infuzide with FDA-approved drugs utilized for the treatment of staphylococcal infections

Drug	MIC (µg/mL) of drug alone	MIC (µg/mL) of Infuzide in presence of drug ("A")	MIC (µg/mL) of drug in presence of Infuzide ("B")	FIC A	FIC B	FIC index	Inference
Ceftazidime	16	1	16	1	1	2	No Interaction
Daptomycin	2	0.5	1	0.5	0.5	1	No Interaction
Gentamicin	0.25	0.5	0.0625	0.5	0.25	0.75	No Interaction^*a*^
Levofloxacin	0.25	1	0.25	1	1	2	No Interaction
Linezolid	2	0.25	0.25	0.25	0.125	0.375	Synergy
Meropenem	0.25	1	0.25	1	1	2	No Interaction
Minocycline	0.25	0.5	0.03125	0.5	0.125	0.625	No Interaction^*a*^
Rifampicin	0.0039	0.5	0.00195	0.5	0.5	1	No Interaction
Vancomycin	2	1	2	1	1	2	No Interaction

^*a*^ Partial synergism.

**Fig 3:**
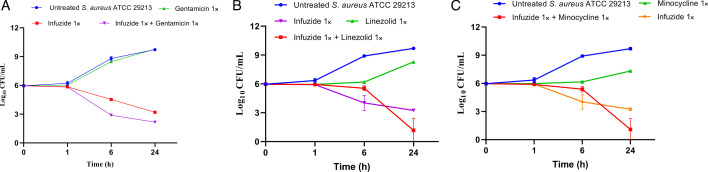
Time-kill kinetics of Infuzide in combination with (**A**) gentamicin, (**B**) linezolid, and (**C**) minocycline against *S. aureus* ATCC 29213 at various time points. Each experiment was performed in triplicate, and the entire experiment was repeated twice. The average values are provided with standard deviations wherever applicable.

**Fig 4:**
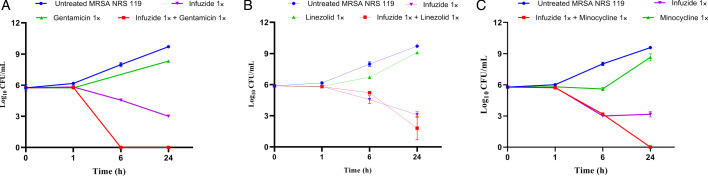
Time-kill kinetics of Infuzide in combination with (**A**) gentamicin, (**B**) linezolid, and (**C**) minocycline against linezolid- and gentamicin-resistant MRSA NRS119 at various time points. Each experiment was performed in triplicate, and the entire experiment was repeated twice. The average values are provided with standard deviations wherever applicable.

In order to confirm if the observed synergy also reflected in reduction in cfu/mL counts, a combination time-kill kinetic analysis was performed against both drug-susceptible *S. aureus* ATCC 29213 (Fig. 3A through C) and MDR MRSA NRS119 (Fig. 4A through C) at 1× MIC of each drug. Against *S. aureus* ATCC 29213, the combination with linezolid and infuzide reduced ~4.8 log_10_ cfu/mL at 24 h, the combination with gentamicin and infuzide reduced ~3.8 log_10_cfu/mL at 24 h, whereas minocycline and infuzide combined reduced ~4.9 log_10_cfu/mL at 24 h compared with the initial colony counts. In all cases, the combination was more potent than either drug alone. A similar trend was observed against MDR MRSA NRS 119 that is resistant to linezolid, gentamicin, and minocycline, where each drug combination with infuzide is more potent than either drug alone and reduces between ~4.2 and 6 log_10_ cfu/mL compared with the initial colony count. The data obtained highlight the potency of Infuzide to be used in combination with protein synthesis inhibitors against infections caused by MDR *S. aureus*.

### Infuzide is highly active against pre-formed *S. aureus* biofilms

**Fig 5:**
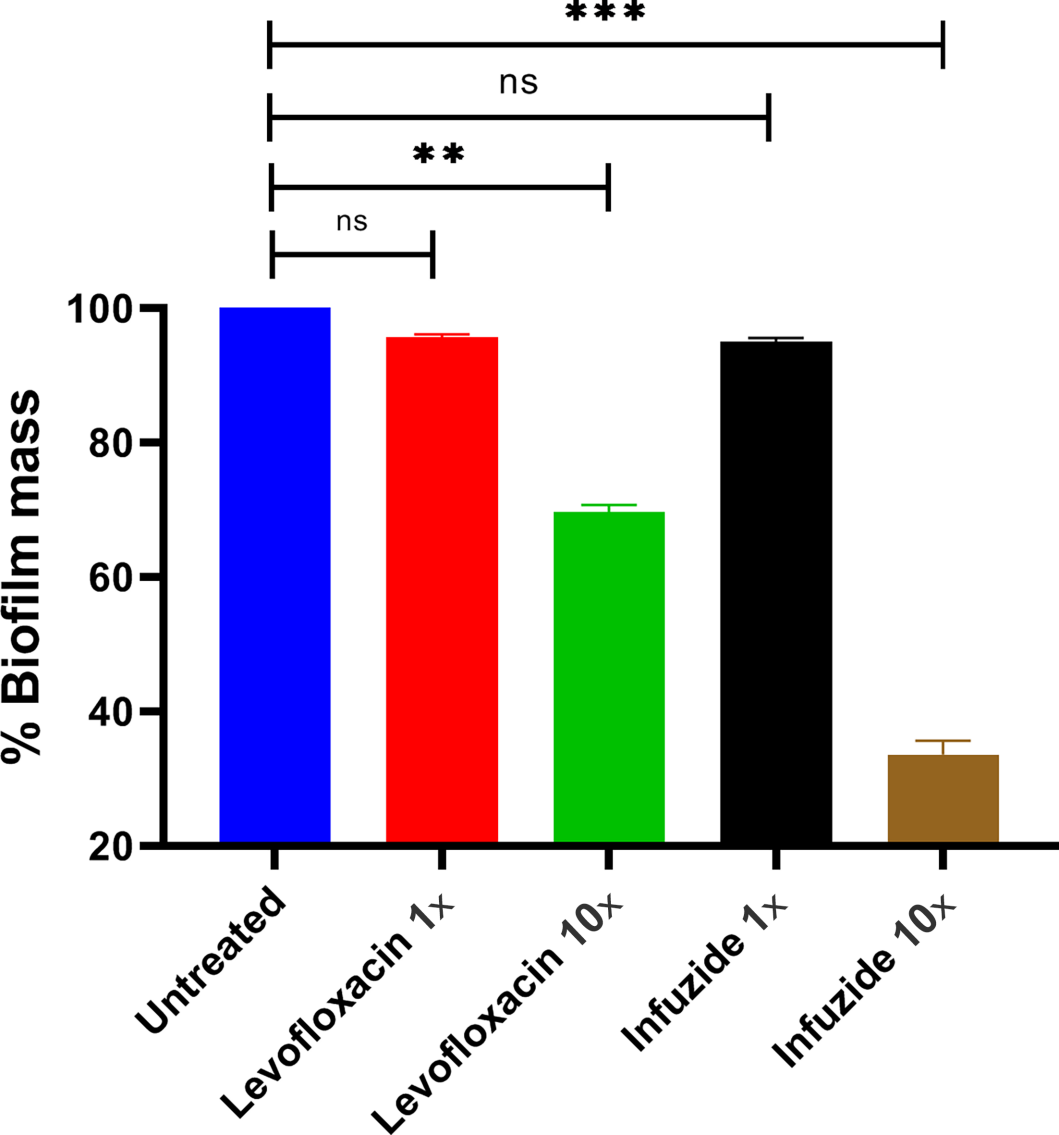
Anti-biofilm activity of Infuzide against *S. aureus* biofilm along with levofloxacin as comparator. The compound significantly reduced the percent biomass of pre-formed bacterial biofilm compared with the control drug. Each experiment was performed in triplicate, and the entire experiment was repeated twice. The average values are provided with standard deviations wherever applicable. * Indicates *P* < 0.5, ** indicates *P* < 0.05, and *** indicates *P* < 0.005.

Under highly adverse conditions such as nutrient deficiency or under assault by host immune response, planktonic bacteria often lead to the formation of multi-cellular communities in an extracellular polysaccharide matrix called biofilms. This transition allows the pathogens to evade the immune response as well as the action of antimicrobial agents. Indeed, it has been shown that bacteria in biofilm state exhibit multi-fold resistance to various immune effectors and antimicrobials, leading to delayed wound healing with consequent increase in hospitalization-associated costs and negatively impacting morbidity and mortality (22, 23). In this context, it is essential to test the efficacy of Infuzide and levofloxacin as comparators against bacteria in biofilm stage, and the results are depicted in Fig. 5. Infuzide at 1× MIC led to ~5% reduction in biofilm mass, which was comparable with that of levofloxacin (~5%), whereas at 10× MIC, Infuzide reduced ~68% of biofilm mass, whereas levofloxacin at the same concentration reduced only ~31%. Thus, Infuzide is twice as effective against pre-formed bacterial biofilm than levofloxacin.

### Infuzide is potently active against intracellular *S. aureus*

**Fig 6:**
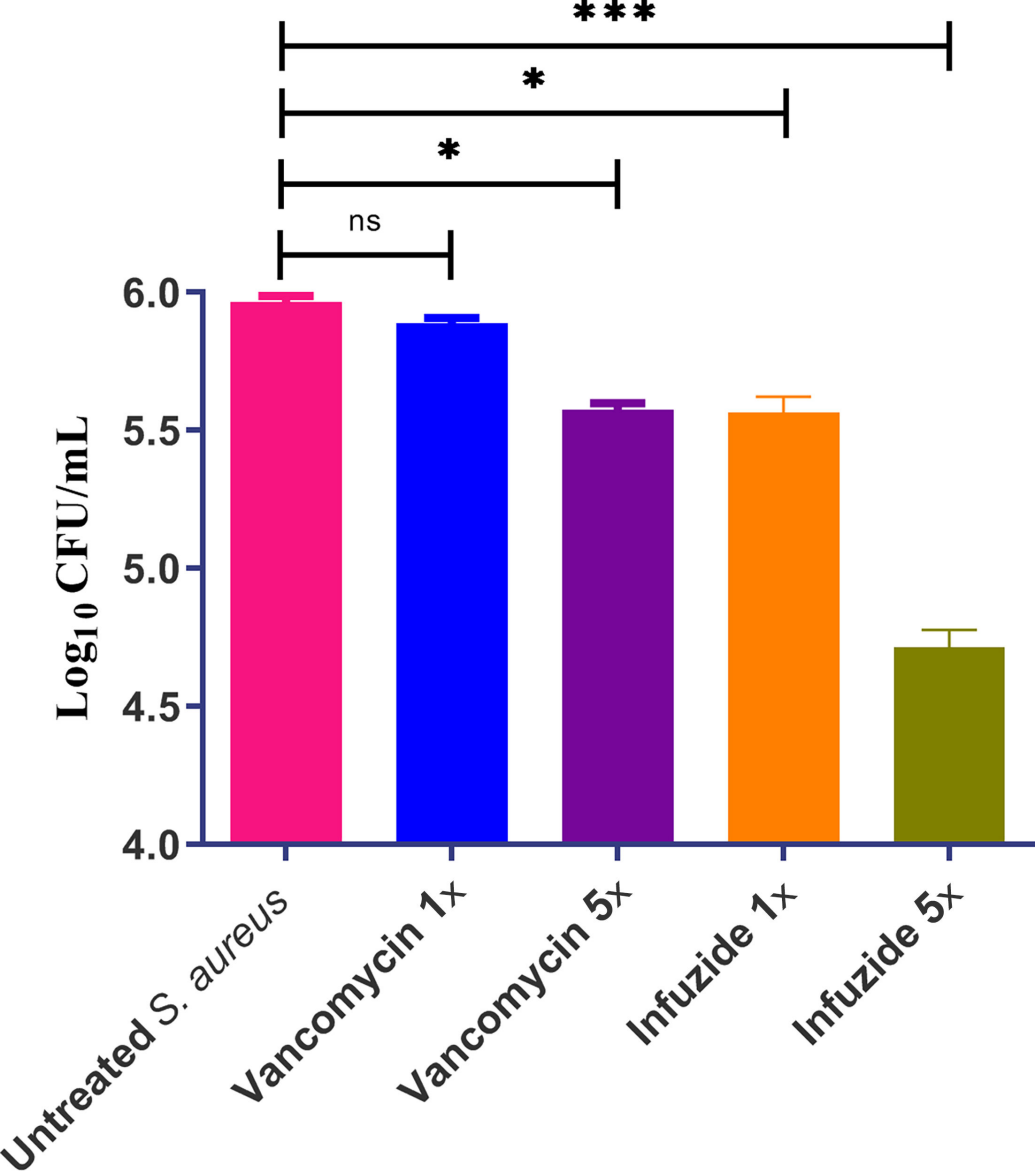
Activity of Infuzide against intracellular *S. aureus* ATCC 29213 along with vancomycin in J774 macrophages at multiple concentrations. Each experiment was performed in triplicate, and the entire experiment was repeated twice. The average values are provided with standard deviations wherever applicable. * Indicates *P* 0.5, ** indicates *P* < 0.05, and *** indicates *P* < 0.005.

Although primarily recognized as an extracellular pathogen, *S. aureus* is known to invade and establish a persistent intracellular infection in phagocytic and non-phagocytic eukaryotic cells. This intracellular niche provides a respite from host immune response as well as from antimicrobials (24). Thus, the activity of Infuzide and vancomycin as comparator was determined against intracellular *S. aureus* in J774 murine macrophages, and the data are shown in Fig. 6. Infuzide at 1× MIC mirrors the time-kill kinetics and reduces ~0.4 log_10_ cfu/mL, which is comparable with vancomycin at 5× MIC. On the other hand, Infuzide at 5× MIC leads to a reduction of ~1.25 log_10_ cfu/mL, which is highly significant. Thus, Infuzide out-competes vancomycin in its activity against intracellular *S. aureus*. This activity is noteworthy, as it demonstrates that Infuzide is not only active against rapidly growing planktonic bacteria but also active against bacteria in altered metabolic states such as in biofilm and intracellular niches. Additionally, it also demonstrates the paucity of FDA-approved treatment options against bacteria in altered metabolic states.

### Infuzide does not select for resistance in *S. aureus*, determination of frequency of resistance (FOR), and mutant prevention concentration (MPC)

**Fig 7:**
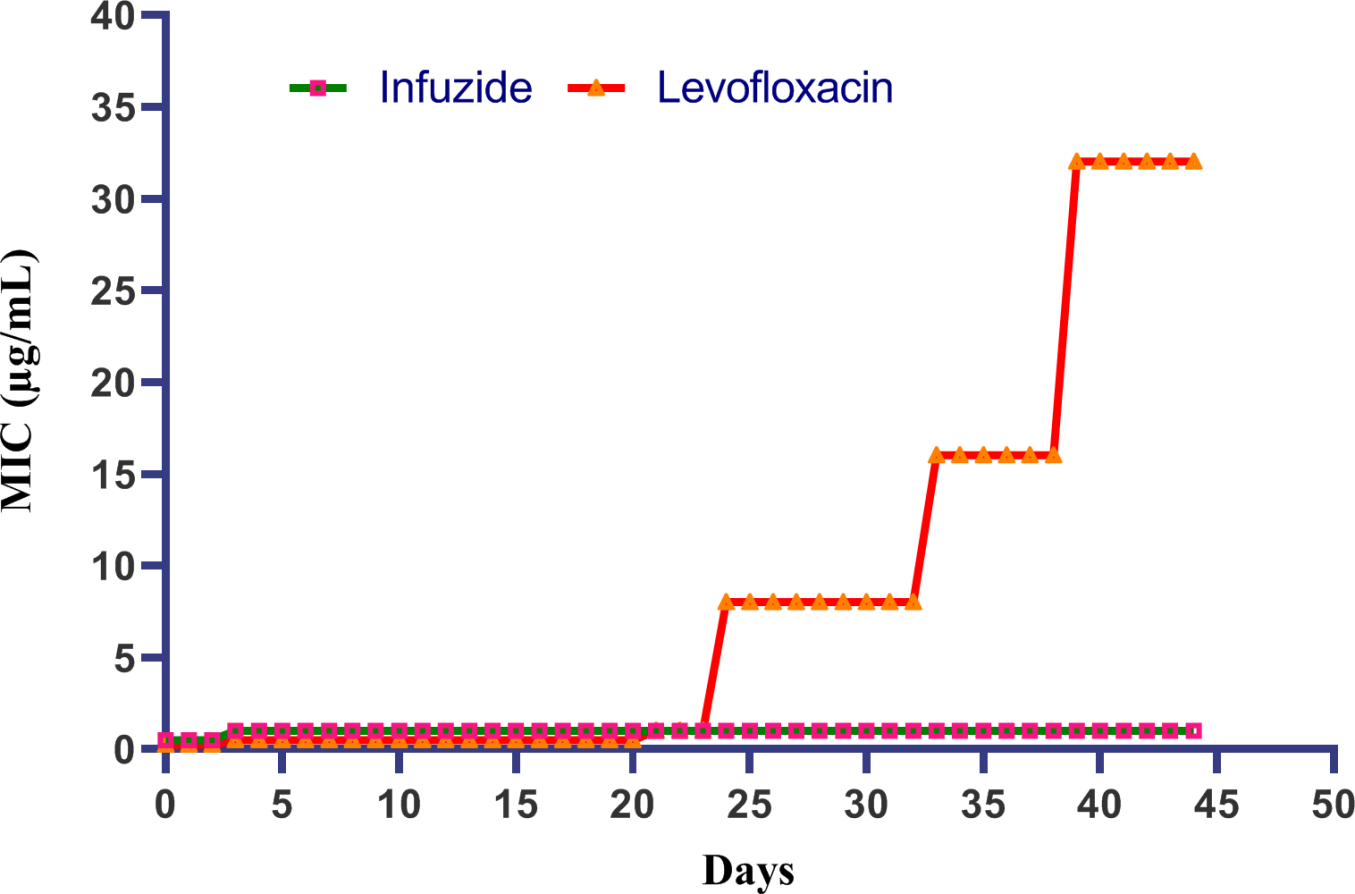
Resistance induction studies of Infuzide against *S. aureus* ATCC 29213. Each experiment was performed in triplicate, and the entire experiment was repeated twice. The average values are provided with standard deviations wherever applicable.

AMR is one of the biggest healthcare challenges affecting medical systems worldwide. In fact, there is hardly any antimicrobial to which the pathogens have not generated resistance to, thus effectively reducing the armamentarium available for the treatment of serious infections. Thus, it is imperative to determine the resistance induction capacity of Infuzide against *S. aureus,* and the data are shown in Fig. 7. Levofloxacin, a DNA gyrase inhibitor, was used as a comparator and induced a characteristic step-wise pattern of resistance with a 128-fold change in MIC after 45 days of sub-MIC exposure. On the same hand, Infuzide induced only 1-fold change in MIC, which is negligible.

Additionally, the frequency of resistance (FOR) was calculated for infuzide and levofloxacin and was very comparable with each other, as well as mutant prevention concentration (MPC) was determined to be 8× MIC for Infuzide and 4× MIC for levofloxacin (Table 6). Taken together, Infuzide is a rapidly bactericidal compound with equipotent activity against multiple clinical, MDR strains expressing various resistance determinants and clinically relevant efflux pumps, along with superior activity against bacteria in various physiological states that does not select for resistance.

**TABLE 6:** Calculation of frequency of resistance and mutant prevention concentration of Infuzide and comparator levofloxacin against *S. aureus* ATCC 29213^*a*^

Drug	MIC (mg/L)	Mutant frequency and fold MIC				Mutant prevention concentration (MPC)
		1×	2×	4×	8×	
Infuzide	1	TNTC	TNTC	1.2 × 10^−6^	0	8× MIC (8 mg/L)
Levofloxacin	0.125	1.5 × 10^−6^	3.2 × 10^−6^	0	0	4× MIC (0.5 mg/L)

^*a*^ TNTC, too numerous to count.

### Infuzide is active against *S. aureus* in neutropenic thigh infection and murine skin infection model

**Fig 8:**
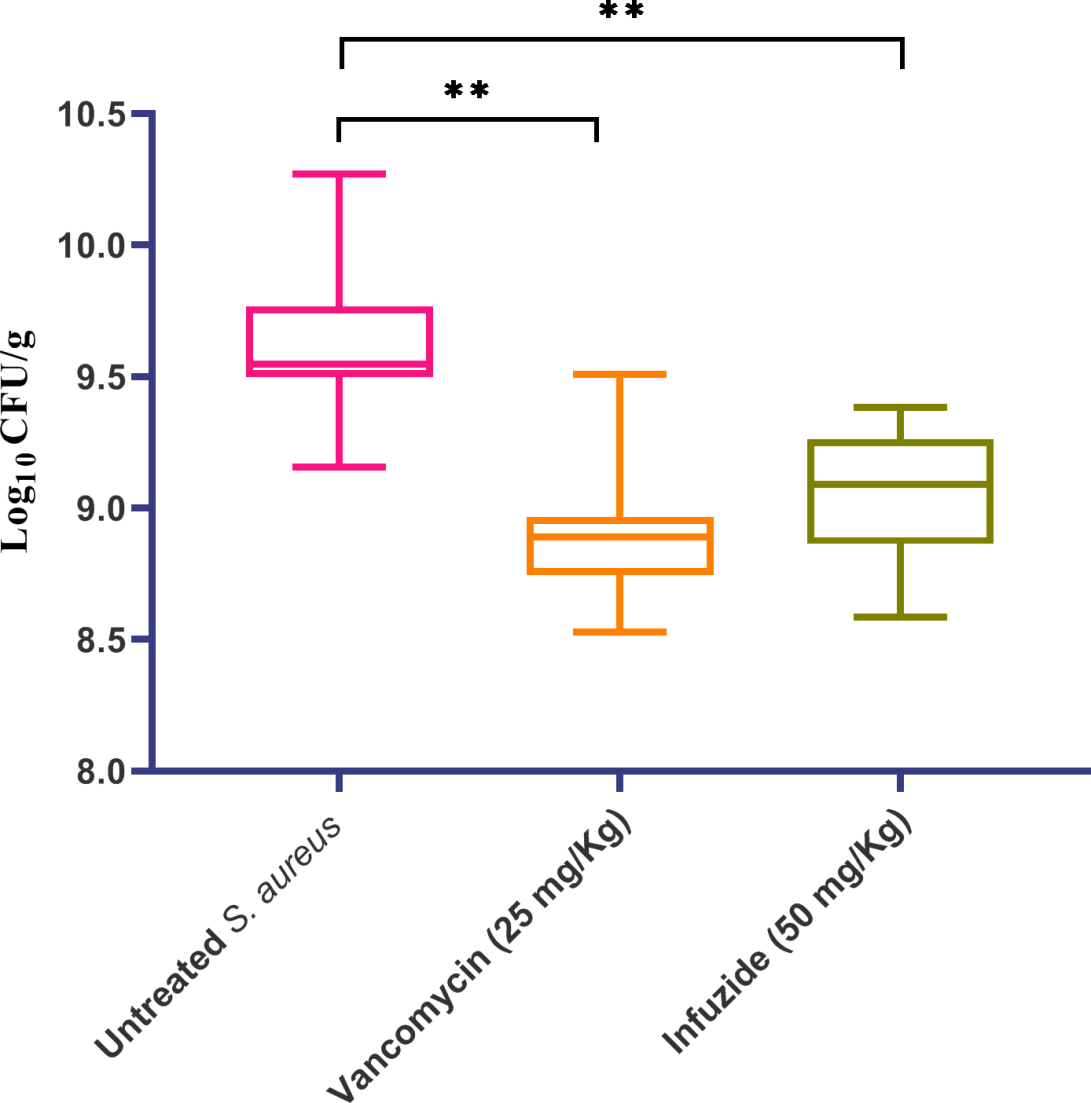
*In vivo* efficacy of Infuzide against *S. aureus* ATCC 29213 in a neutropenic thigh infection model along with vancomycin. Each experiment was performed in triplicate, and the entire experiment was repeated twice. The average values are provided with standard deviations wherever applicable. * Indicates *P* ≥ 0.5, ** indicates *P* < 0.05, and *** indicates *P* < 0.005.

As infuzide exhibited all the desired properties, its maximum tolerated dose (MTD) was determined to be >200 mg/kg with no effect observed on gross growth parameters till 7 days post-administration (Table S1). Following this, Infuzide was tested in the neutropenic thigh infection model of *S. aureus* to determine if the excellent *in vitro* activity translates *in vivo* as well. The neutropenic thigh infection model is a standard model used to determine initial antimicrobial activity (25). The *in vivo* potential of Infuzide (50 mg/Kg) was tested along with vancomycin (25 mg/Kg) as a comparator, and the data are depicted in Fig. 8. As is visualized, treatment with Infuzide led to a ~ 0.65 log_10_ cfu/mL reduction, which is comparable with ~0.7 log_10_cfu/mL reduction upon treatment with vancomycin compared with untreated. This provides the first proof that the excellent *in vitro* MIC exhibited by Infuzide is translating into *in vivo* activity.

**Fig 9:**
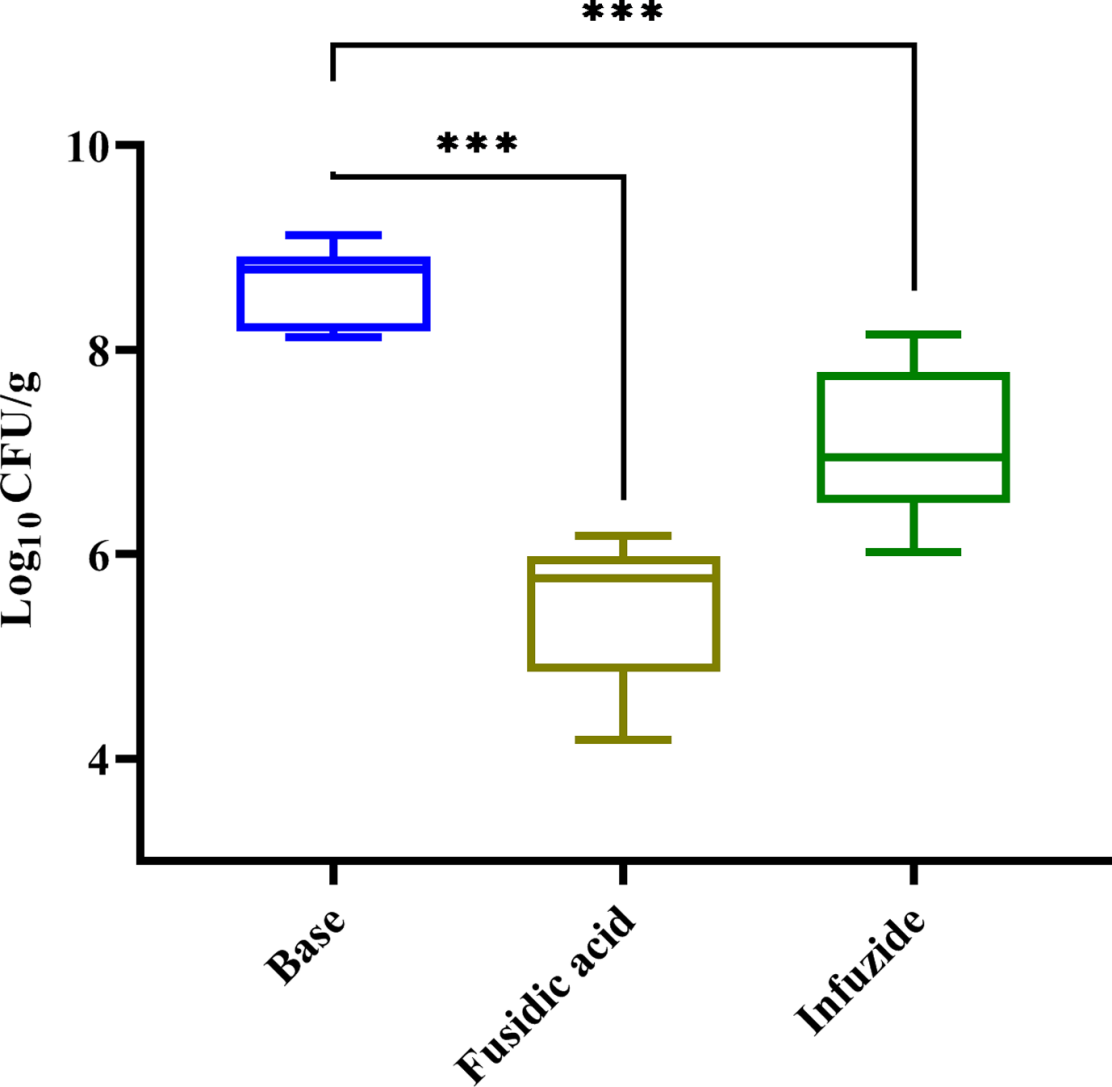
*In vivo* efficacy of Infuzide (2**%**) against *S. aureus* ATCC 29213 in murine superficial skin infection model along with fusidic acid (2%) as control. Each experiment was performed in triplicate, and the entire experiment was repeated twice. The average values are provided with standard deviations wherever applicable. * Indicates *P* ≥ 0.5, ** indicates *P* < 0.05, and *** indicates *P* < 0.005.

In the next step, as *S. aureus* is responsible for commonly occurring to life-threatening skin and skin structure infections (SSTI) in both healthcare and community settings (1), we tested the potential of Infuzide along with fusidic acid as a comparator against *S. aureus* ATCC 29213 in the murine skin infection model, and the data are shown in Fig. 9 (26). When Infuzide was applied as a 2% formulation twice daily for 5 days, it led to a reduction of ~1.6 log_10_ cfu/mL, whereas treatment with fusidic acid (2%) reduced ~3.2 log_10_cfu/mL compared with infected mice treated with just the vehicle. This experiment demonstrates the potential of Infuzide to be utilized for the treatment of SSTI caused by *S. aureus*.

**Fig 10:**
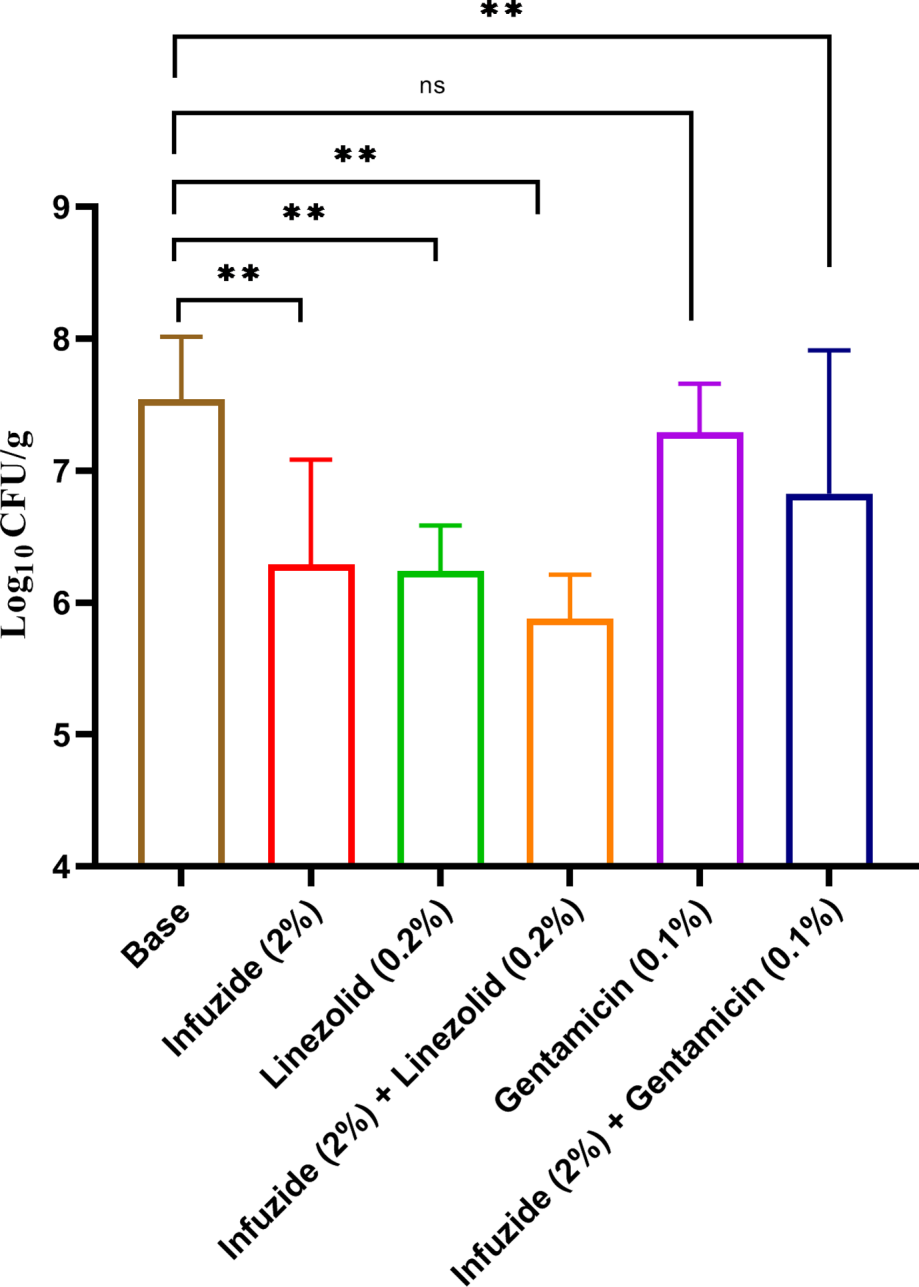
*In vivo* efficacy of Infuzide against gentamicin and linezolid-resistant MRSA NRS119 in murine superficial skin infection model along with comparators. Each experiment was performed in triplicate, and the entire experiment was repeated twice. The average values are provided with standard deviations wherever applicable. * Indicates *P* ≥ 0.5, ** indicates *P* < 0.05, and *** indicates *P* < 0.005.

Taking this further, we wished to test the potential of *in vitro* identified synergistic drug combinations with Infuzide against MDR MRSA NRS 119 in the murine skin infection model, and the results are shown in Fig. 10. As is seen, the combination of Infuzide (2%) and linezolid (0.2%) led to a reduction of ~1.66 log_10_cfu/mL, which is more than that exhibited by individual drugs compared with animals treated with vehicle. Similarly, the combination of Infuzide (2%) and gentamicin (0.1%) led to a reduction of ~0.7 log_10_cfu/mL, which is more than that exhibited by gentamicin but not Infuzide and compared with animals treated with vehicle. Taken together, Infuzide exhibits potent *in vivo* activity, both in the neutropenic thigh infection model and murine skin infection model, that too against both drug-susceptible *S. aureus* ATCC 29213 and MDR MRSA NRS 119.

Taken together, Infuzide exhibits all the desired characteristics needed for a comprehensive anti-staphylococcal: broad equi-potent activity against various MDR *S. aureus* and *Enterococcus*, concentration-dependent bactericidal activity, no induction of resistance, better than SoC against bacterial biofilms, and intracellular pathogens with powerful activity in neutropenic thigh and murine skin infection model. Combined with the fact that the structurally infuzide does not resemble any FDA-approved drug, it highlights the potential of this scaffold against gram-positive WHO high-priority pathogens.

## MATERIALS AND METHODS

### Growth media and reagents

All bacterial media and supplements, including Mueller–Hinton cation supplemented broth II (MHBII), Mueller–Hinton agar (MHA), and tryptic soy broth (TSB), were purchased from Becton-Dickinson (Franklin Lakes, NJ, USA). All other chemicals and antibiotics were procured from Sigma-Aldrich (St. Louis, MO, USA). While performing the experiments, all the relevant guidelines and regulations were followed.

### Bacterial strains and conditions used for the experiments

We have checked the activity of infuzide against *Enterococcus* sp, *Enterobacter* sp, *E. coli* ATCC 25922, *S. aureus* ATCC 29213, *K. pneumoniae* BAA 1705, *A. baumannii* BAA 1605, and *P. aeruginosa* ATCC 27853. The screening further includes the drug-resistant clinical strains of *S. aureus* and *Enterococcus* sp., including those resistant to vancomycin and other clinically utilized antibiotics. These strains were procured from Biodefense and Emerging Infections Research Resources Repository/Network on Antimicrobial Resistance in *Staphylococcus aureus*/American Type Culture Collection (BEI/NARSA/ATCC, USA) and routinely cultivated on MHA and MHBII. For primary culture or initial culture, we have taken one single colony from the MHA plate and inoculated it in MHBII and incubated it at 37°C overnight in a shaking incubator.

### Antibiotic susceptibility testing

Antibiotic susceptibility testing of hit molecules was performed according to CLSI guidelines using the broth microdilution assay (27); 10 mg/mL stock solutions of test compounds were prepared in DMSO. Bacterial cultures were inoculated in MHBII, and optical density (OD) was measured at 600 nm, followed by dilution to achieve ~10^6^ CFU/mL. The anti-bacterial activity of the compounds was tested at concentrations ranging from 64–0.5 mg/L in a 2-fold serial diluted fashion, with 2.5 µL of each concentration added to the well of a 96-well round bottom microtiter plate. Later, 97.5 µL of bacterial suspension was added to each well containing either test compound or appropriate controls. The plates were incubated at 37°C for 18–24 h, following which the MIC was determined. The MIC is defined as the lowest concentration of the compound at which there is an absence of visible bacterial growth. For each test compound, MIC determinations were carried out independently three times using duplicate samples.

### PMBN assay

The MIC of Infuzide against *E. coli* ATCC 25922 and *A. baumannii* BAA-1605 was determined in the presence of 10 mg/L Polymyxin B nonapeptide (PMBN) in a culture broth according to the method utilized for MIC determination (27).

### Cell cytotoxicity

Cell toxicity was performed against Vero, NIH-3T3, and J774 cell lines using the MTT assay (28), and ~10^3^ cells/well were seeded in 96-well flat bottom plates and incubated at 37°C in 5% CO_2_ atmosphere. After 24 h, the infuzide was added ranging from 200 to 1 µg/mL concentration for NIH-3T3 cells, 100–1 µg/mL for Vero, J774 cells, and incubated for an additional 72 h. After incubation, 20 µL MTT was added to each well, and the plates were further incubated at 37°C for 4 h. The residual medium was then discarded, 0.1 mL of DMSO was added to solubilize the formazan crystals, and O. D. was taken at 575 nm. The CC_50_ is defined as the lowest concentration of the compound killing 50% of the eukaryotic cells. Doxorubicin was used as a positive control, and each experiment was repeated in triplicate.

### Hemolysis

The compound was assessed for its hemolytic activity. For this, fresh human blood was collected from a healthy volunteer in an anticoagulant-containing vial. After serum separation, the RBCs collected were washed with 1× PBS by centrifuging the cells at 700 *× g* for 10 min and resuspending the cells in 1× PBS. A 4% (vol/vol) suspension of hRBCs in PBS was then subjected to 100, 50, 25, and 12.5 mg/L concentrations of compound or the controls in a final volume of 0.2 mL. The plates were then incubated at 37°C for 45 min. After incubation, the samples were centrifuged at 700 *× g* for 10 min, and the absorbance (Abs Sample) of the released hemoglobin in the supernatant was measured at 540 nm. hRBCs in PBS (Abs Blank) and in 0.2% (vol/vol) Triton X-100 (Abs Triton) were taken as negative and positive controls, respectively. The percentage of hemolysis was calculated as: Percentage of hemolysis = [(AbsSample-AbsBlank)/(AbsTriton-AbsBlank)]*100 (29).

### Drug-drug interaction study

Interaction of Infuzide with FDA-approved drugs was tested by the checkerboard method. Serial 2-fold dilutions of each drug were freshly prepared prior to testing. Infuzide was 2-fold diluted along the abscissa, whereas the antibiotics were serially diluted along the ordinate in a 96-well microtiter plate; 95 µL of ~10^5^ CFU/mL was added to each well, and plates were incubated at 37°C for 24 h. After the incubation, the ΣFICs (fractional inhibitory concentrations) were calculated as follows: ΣFIC = FIC A + FIC B, where FIC A is the MIC of drug A in the combination/MIC of drug A alone and FIC B is the MIC of drug B in the combination/MIC of drug B alone. The combination is considered synergistic when the ∑FIC is ≤0.5, indifferent when the ∑FIC is >0.5–4, and antagonistic when the ∑FIC is >4 (30).

### Time-kill kinetics

The presence or absence of bactericidal activity was assessed by the time-kill method as described earlier (31). Briefly, *S.* aureus ATCC 29213 was diluted to ~10^6^ CFU/mL in MHBII and treated with 1×, 5×, and 10× MIC of Infuzide and vancomycin and incubated at 37°C with shaking for 24 h; 100 µL of the samples was collected at 0, 1, 6, and 24 h, serially diluted in PBS, and plated on MHA, followed by incubation at 37°C for 18–20 h. The kill curves were constructed by counting the colonies from plates and plotting the CFU/mL of surviving bacteria at each time point in the presence and absence of compound. Each experiment was repeated three times in duplicate, and the mean data were plotted.

### Selection of resistant mutant

*S. aureus* ATCC 2913 was serially passaged in the presence of 0.5× MIC of Infuzide and levofloxacin as a positive control and freshly sub-cultured every 24 h (32). MIC was tested every 3 days for each compound-induced culture with wild-type *S. aureus* ATCC 29213 as a control.

### *In vitro* determination of resistant frequency

*In vitro* spontaneous resistance frequency and mutant prevention concentration of infuzide and levofloxacin were calculated as per previous protocol (33–35). *S. aureus* ATCC 29213 (~10^6^ CFU) was spread on Mueller Hinton agar plates containing 1× to 16× MIC of Infuzide and levofloxacin and incubated at 37^°^C. The plates were observed daily from the 1st to the 3rd day for the appearance of colonies. The resistant colonies from these plates were counted and divided by the initial CFU plated. MPC is the concentration of the drug where no colonies were observed.

### PAE

To determine the PAE of Infuzide, an overnight culture of *S. aureus* ATCC 29213 was diluted in MHBII ~ 10^5^ CFU/mL and exposed to 1× and 10× MIC of vancomycin and Infuzide and incubated at 37°C for 1 h. Following the end of the incubation period, the culture was centrifuged and washed two times with pre-warmed TSB to remove any traces of antibiotics. Finally, the cells were re-suspended in drug-free TSB and incubated further at 37°C. Samples were taken after every 1 h, serially diluted, and plated on TSA for enumeration of CFU. The PAE was calculated as PAE = T – C; where T is referred to the difference in time required for 1 log_10_ increase in CFU versus CFU observed immediately after removal of drug, and C is the time difference in a similarly treated drug-free control (36).

### Biofilm assay

As described previously, in 1% TSB, *S. aureus* ATCC 29213 was cultured overnight at 37°C and 180 rpm (37). The overnight culture was diluted at 1:100 in TSB supplemented with 1% glucose, and 0.2 mL/well with 1× and 10× concentration of Infuzide, vancomycin, and levofloxacin was transferred into 96-well polystyrene flat-bottom tissue culture plates. To increase biofilm formation, low oxygen concentration was maintained and kept at 37°C for 48 h. After 48 h, media were decanted, and the plates were rinsed gently three times with 1× PBS (pH = 7.4) to remove the planktonic bacteria. After that, the biofilm was fixed by incubating the plate at 60°C for 1 h. After fixing, the biofilm is stained with 0.06% crystal violet for 10 min, rinsed with PBS, and dried at room temperature. The bound crystal violet was eluted with 30% acetic acid (0.2 mL) for biofilm quantification. Absorbance was taken on a microtiter plate reader at 600 nm for biofilm quantification.

### Intracellular killing with Infuzide

The J774.A1 mouse macrophage cell line was seeded at 50,000 cells/well in a 12-well tissue culture plate and was infected with *S. aureus* ATCC 29213 for 1 h at a MOI of 1:100. After infection, cells were washed with 1× PBS (pH 7.4) to remove extracellular bacteria, and the wells were replaced by RPMI medium containing different concentrations of drugs containing lysostaphin at 10 units per 0.5 mL media, which was utilized to prevent extracellular bacterial growth. The plates incubated for 24 h at 37°C in a 5% CO_2_ incubator. Following the incubation, the cells were washed 3 times with 1× PBS (pH 7.4) and lysed with RIPA buffer (#89901 Thermo). The cell lysate was serially diluted, plated on TSA, and incubated for 24 h at 37°C for enumeration of colony-forming units (CFU) (37).

### Maximum tolerability dose study

For the MTD study, 5- to 6-week-old Swiss mice of 22–25 gm were randomly distributed into groups, with 3 mice assigned to each group. After 24 h of acclimatization, the mice were administered different concentrations of the compound as required (25, 50, 100, and 200 mg/kg of body weight). The compound was given as a single dose administered intra-peritoneally, whereas one group was taken as a control group with no drug administration. The animals under study were continuously monitored for the change in parameters such as weight, feed, or fur quality after the compound administration. Additionally, the survival of the animals was also studied on compound treatment. The experiment was carried out for 7 days, with the mentioned parameters being recorded on a daily basis. The maximum tolerated dose (MTD) is commonly estimated to be the maximum dose that can be administered for the duration of a defined period that will not compromise the survival of the animals (38).

### Murine thigh infection model

Efficacy of infuzide to reduce bacterial burden *in vivo* was determined by using the murine neutropenic *S. aureus* ATCC 29213 thigh infection model. Briefly, male Swiss mice weighing (~22–25gm) were used throughout the study and were rendered neutropenic by a series of cyclophosphamide injections given intraperitoneally (IP) 4 day and 1 day before infection. This was followed by an injection of *S. aureus* ATCC 29213 in the right thigh of mice to establish infection. After 2 h of infection, infuzide and vancomycin at different doses were injected IP into mice twice at an interval of 4 h between injections. The control animals were administered saline in the same volume and frequency as those receiving treatment. After 24 h, the mice were sacrificed, thigh tissue was collected from the animals, weighed, homogenized in 5 mL of saline, and serially diluted followed by plating on MHA plates for CFU determination. After incubation for 18–24 h at 37°C, the CFU were enumerated. Each experiment was repeated three times in duplicate, and the mean data are plotted (31).

### Murine skin infection model

*In vivo* study of infuzide has been done by superficial skin infection model as described in reference with small change (37). Briefly, male Swiss mice 4–6 weeks old, weighing ∼22–25 g, were used throughout the study and were caged alone in an individually vented cage (IVC) to check cross-contamination and to maintain aseptic conditions throughout the experiment. Ketamine and xylazine were prepared in distilled water, 40 and 8 mg/kg of body weight, respectively, and injected IP as a mixture of 100 µL in each mouse for anesthesia. Then, we proceeded with the removal of fur by applying depilatory cream; the area was cleaned with the help of sterile distilled water, and ~2 cm^2^ of skin area was scratched until it became visibly damaged and was characterized by reddening and glistening without any bleeding. For bacterial infection, a 10 µL droplet containing 10^7^ CFU/ml of *S. aureus* ATCC 29213 was applied to the reddened area of skin. To confirm the infection, post 4 h, untreated mice were sacrificed. Various dilutions were plated on the TSA plate at the exact time dosing of the treatment groups, that is, 2% fusidic acid (positive control), 2% infuzide, and base (vehicle) was started, followed by a second dose post 16 h from the first dose and, henceforth, drug regimen comprised of twice daily application (in the morning and the evening, with an 8 h interval) for 4 days. The mice were regularly treated with 25–30 mg each of 2% fusidic acid (LEO Pharma, Ballerup, Denmark) and 2% infuzide and base (vehicle). All groups were sacrificed post-18 h of the last dose to avoid the carryover effects of the treatment group. Around ∼2 cm^2^ of wounded skin post-sacrifice were excised and homogenized with 500 µL phosphate-buffered saline (1× PBS) in 2 mL of MP tissue grinding Lysing Matrix F tubes using MP FastPrep-24 set at 4.0 M/S for 30 s (three cycles). The dilutions were plated posthomogenization on TSA plates, followed by overnight incubation at 37°C to determine the bacterial CFU count in each treatment group. Each experiment was repeated three times in duplicate, and the mean and SEM of log_10_ CFU/g were plotted (32).

### Statistical analysis

Statistical analysis was performed using GraphPad Prism 8.0.2 software (GraphPad Software, La Jolla, CA, USA). Comparison between three or more groups was analyzed using a one-way analysis of variance with Dunnett’s multiple comparison test. *P* values < 0.05 were considered significant.

## SUPPLEMENTAL MATERIAL

Supplemental material spectrum.00279-25-s0001.docx Fig. S1 and S2; Table S1.

ASM does not own the copyrights to Supplemental Material that may be linked to, or accessed through, an article. The authors have granted ASM a non-exclusive, world-wide license to publish the Supplemental Material files. Please contact the corresponding author directly for reuse.
