# Supplementary material for: Comprehensive biological evaluation of infuzide as a potent antimicrobial, alone and in combination with gentamicin, linezolid, and minocycline targeting MDR Staphylococcus aureus and Enterococcus sp
Source: Microbiol Spectr. 2025 Jun 2;13(7):e00279-25. doi: 10.1128/spectrum.00279-25 (PMC12211062; doi:10.1128/spectrum.00279-25)
Supplement: Supplemental material — Fig. S1 and S2; Table S1. [file spectrum.00279-25-s0001.docx]

**Fig S1:** Stability studies of Infuzide at different time points T-0, T=36h and T=72h analyzed byanalytical reverse-phase chromatography. A solution of Infuzide (3mg) in MeOH + 0.1 % TFA (0.5ml) was injected at T=0 min; T=30h, T=54h and T=25 day in a XBridge C18 5 μm column and representatives are shown below at 300, 330 and 400 nm.

**300 nm**

T = 0 (eluant water / methanol + 0.1% TFA)

T = 30h (eluant water / acetonitrile + 0.1% TFA)

T = 54h

T = 25d

**330 nm**

T = 0 (eluant water / methanol + 0.1% TFA)

T = 30h (eluant water / acetonitrile + 0.1% TFA)

T = 54h (eluant water / acetonitrile + 0.1% TFA)

T = 25d (eluant water / acetonitrile + 0.1% TFA)

**400 nm**

T = 0 (eluant water / methanol + 0.1% TFA)

T = 30h (eluant water / acetonitrile + 0.1% TFA)

T = 54h (eluant water / acetonitrile + 0.1% TFA)

T = 25d (eluant water / acetonitrile + 0.1% TFA)

Parameters.

Solvent A = water + 0.1 % TFA

Solvent B = methanol + 0.1% TFA (Time 0) or acetonitrile + 0.1% TFA (0h; 30h, 54h and 25 days)

Elution conditions: 90% A / 10% B for 2 minutes, then gradient up to 95% B over 20 minutes, then 95% B for 4 minutes (total 26 minutes).

Column Analytical equipment: Agilent technologies 1200 series

ColumnXBridge C18 5 µm, diameter 4.6 mm, length 150 mm.

Injection volume: 10 µL (solution in methanol + 0.1 % TFA)

**Fig S2:Hemolysis potential of Infuzide as tested against human RBC**. Each experiment was performed in triplicate and the entire experiment was repeated twice. The average values are provided with standard deviations wherever applicable. HC_50_ stands for concentration to lyse 50% of the red blood cells and Triton X-100 is used as a positive control.


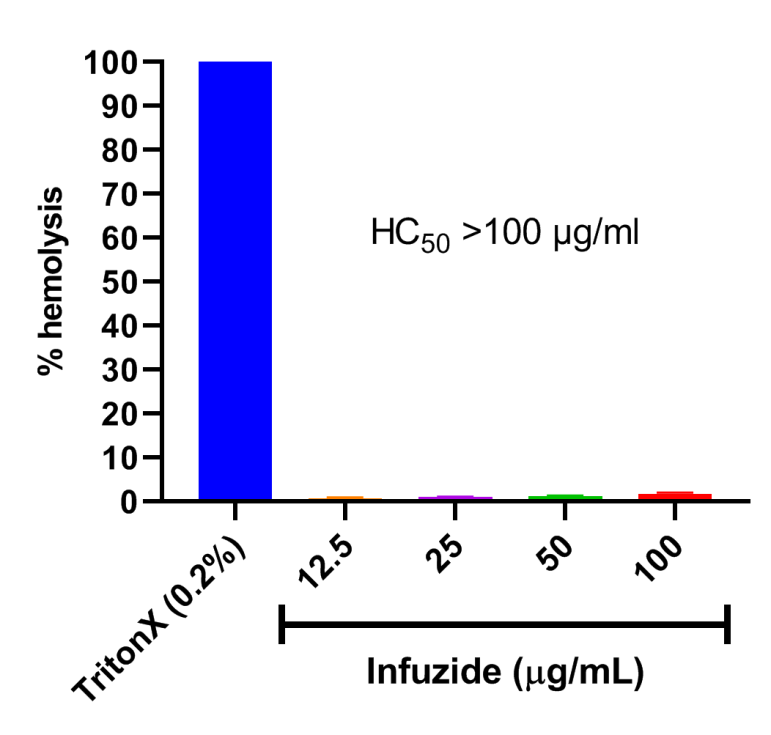


**Table S1:** Survivability of mice injected with single dose of **Infuzide**

| **Day** | **Untreated** | **Treated with MB-7** | | | |
| --- | --- | --- | --- | --- | --- |
|  |  | **25 mg/kg** | **50 mg/kg** | **100 mg/kg** | **200 mg/kg** |
| **0** | 3 | 3 | 3 | 3 | 3 |
| **1** | 3 | 3 | 3 | 3 | 3 |
| **2** | 3 | 3 | 3 | 3 | 3 |
| **3** | 3 | 3 | 3 | 3 | 3 |
| **4** | 3 | 3 | 3 | 3 | 3 |
| **5** | 3 | 3 | 3 | 3 | 3 |
| **6** | 3 | 3 | 3 | 3 | 3 |
